# High-resolution MRI findings in patients with capsular warning syndrome

**DOI:** 10.1186/1471-2377-14-16

**Published:** 2014-01-20

**Authors:** Lixin Zhou, Jun Ni, Weihai Xu, Ming Yao, Bin Peng, Mingli Li, Liying Cui

**Affiliations:** 1 Department of Neurology, Peking Union Medical College Hospital and Chinese Academy of Medical Science, Shuai Fu Yuan 1#, Dong Cheng District, Beijing 100730, China; 2 Department of Radiology, Peking Union Medical College Hospital and Chinese Academy of Medical Science, Shuai Fu Yuan 1#, Dong Cheng District, Beijing 100730, China

**Keywords:** Capsular warning syndrome, High-resolution MRI, Middle cerebral artery, Atherosclerotic plaque

## Abstract

**Background:**

Capsular warning syndrome (CWS) is rare (1.5% of TIA presentations) but has a poor prognosis (7-day stroke risk of 60%). Up to date, the exact pathogenic mechanism of CWS has not been fully understood. We report the clinical presentations and high-resolution MRI (HR MRI) findings of two cases with capsular warning symptoms.

**Case presentation:**

Case 1 was a 63-year-old man with a history of hypertension with recurrent episodes of left hemiparesis and dysarthria lasting 10 ~ 30 minutes. Case 2 was a 54-year-old woman with repetitive episodes of transient left hemiparesis and dysarthria lasting about 10 minutes. Capsular infarctions on DWI were demonstrated in the territory of a lenticulostriate artery in both 2 patients. HR MRI disclosed atherosclerotic plaques on the ventral wall of the MCA where enticulostriate arteries were arisen from, although traditional digital subtraction angiography showed normal. Aggressive medical therapy with dual antithrombotic agents and statin was effective in these two cases.

**Conclusion:**

Our HR MRI data offer an insight into the pathophysiology of CWS which might be caused by atherosclerotic plaque in non-stenotic MCA wall. HR MRI might be a useful modality for characterizing atherosclerotic plaques in the MCA and detecting the pathophysiology of the CWS.

## Background

The term of capsular warning syndrome (CWS) was first described in 1993 in patients who presented repeated stereotyped episodes of subcortical transient ischemic attacks [1]. It is a popular notion that the syndrome is associated with a high risk of developing a completed stroke [2,3]. Up to date, little is known about pathogenic mechanisms and the role of atherosclerotic disease of the middle cerebral artery (MCA) in CWS.

A few recent studies have confirmed the feasibility of using high-resolution magnetic resonance imaging (HR MRI) to depict the MCA wall and plaques in vivo [4,5]. We report two cases with typical CWS. High-resolution MRI demonstrated small atherosclerotic plaques on the ventral wall of proximal MCA, although digital and MR angiography showed no abnormalities. According to the findings of HR MRI, possible pathogenic mechanisms of CWS are discussed.

## Case presentation

### Case 1

**Figure 1:**
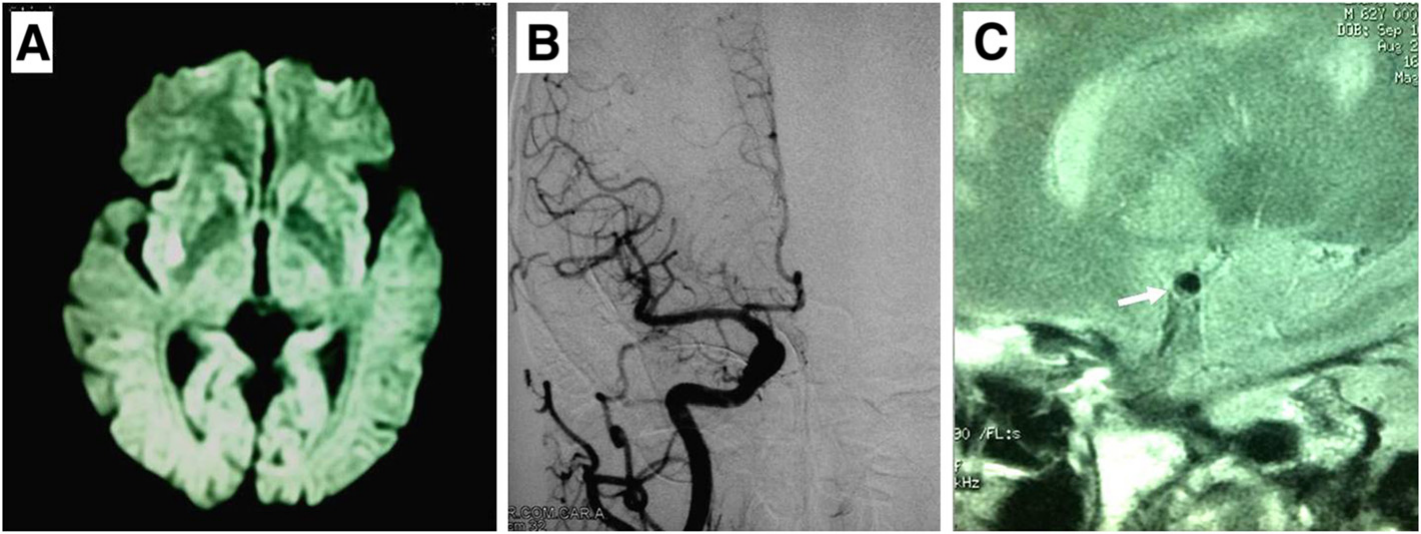
**MRI, DSA and HR MRI findings of Case 1. (A)** MRI diffusion weighted image shows a hypersensitive lesion on right putamen with rostral extension to the corona radiates. **(B)** Digital angiography of the right MCA shows normal. **(C)** High-resolution MRI of the MCA demonstrates an atherosclerotic plaque (arrow) on the frontal wall of the right proximal MCA.

A 63-year-old man with a history of hypertension was seen with recurrent episodes of left hemiparesis and dysarthria lasting 10 ~ 15 minutes in the emergency room. His blood pressure was 136/94 mmHg. Neurological examination on admission was unremarkable. Treatment of antithrombotic drug (aspirin 200 mg) and hydration was given immediately. During the first 48 hours hospitalization he experienced 9 times episodes of left hemiparesis, left central type facial palsy, and dysarthria lasting 13 to 36 minutes. His neurologic status fluctuated between 5 and 11 on the National Institutes of Health Stroke Scale (NIHSS) and he couldn’t relieve completely after the last episode with left mild hemiparesis and facial palsy (NIHSS 3). Brain MRI showed an acute infarct on right putamen with rostral extension to the corona radiates (Figure 1A). A transthoracic echocardiogram revealed no cardiac source of embolism. Digital angiography of cerebral vascular showed no significant abnormal (Figure 1B). 3-T high-resolution MRI of bilateral MCA demonstrated an atherosclerotic plaque on the ventral wall of the right proximal MCA causing the vessel lumen irregular (Figure 1C). Medical management then switched to a combination of aspirin (200 mg daily), clopidogrel (75 mg daily) and statin (atorvastatin calcium 20 mg daily). There were no more episodes later. The patient was discharged with mild facial palsy (NIHSS 1) 2 weeks later.

### Case 2

**Figure 2:**
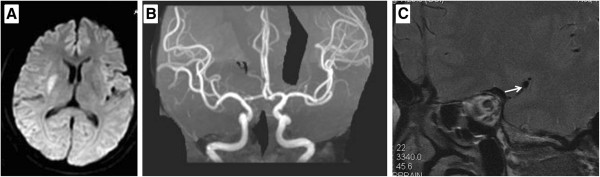
**MRI, MRA and HR MRI findings of Case 2. (A)** MRI diffusion weighted image shows a hypersensitive lesion on posterior limb of internal capsular. **(B)** MR angiography of the MCA is normal. **(C)** High-resolution MRI of MCA indicates the presence of an atherosclerotic plaque on the frontal wall of the right proximal MCA.

A 54-year-old woman developed a sudden onset of left hemiparesis and dysarthria lasting 10 minutes. After 4 times episodes of transient left hemiparesis in next 4 hours, the patient was admitted to our emergency room. She denied previous episodes of transient ischemic attack or stroke. There is no known history of hypertension, diabetes, dyslipidemia, migraine, or heart disease. Her blood pressure was 170/100 mm Hg on admission. Neurological examination disclosed mild left hemiparesis (grade 4). Brain MRI demonstrated an acute right-sided infarct on posterior limb of internal capsular (Figure 2A). Brain MRA showed no significant abnormalities (Figure 2B). High-resolution MRI of MCA indicated the presence of an atherosclerotic plaque on the ventral wall of the right proximal MCA (Figure 2C). The patient had at least two episodes of transient left hemiparesis in the emergency room. Aggressive medical management including a combination of antithrombotic agents of aspirin (200 mg daily) and clopidogrel (75 mg daily), and statin (atorvastatin calcium 20 mg daily) was given immediately. The remainder of the patient’s hospital course was uneventful.

## Discussion

This is the first study to date on HR MRI findings of MCA cross-section in patients with CWS. Capsular infarctions were demonstrated in the territory of a lenticulostriate artery on DWI in both 2 patients. Although cerebral vascular angiography showed no abnormalities, HR MRI disclosed atherosclerotic plaques on the ventral wall of MCA where enticulostriate arteries were arisen from. These HR MRI findings suggested that MCA atherosclerotic disease may play an important role in pathophysiology of the CWS in our cases.

The ‘capsular warning syndrome’, a term introduced by Donnan et al., denotes repetitive transient ischemia attacks causing stereotyped unilateral motor, sensory, or sensorimotor deficits that simultaneously affect the face, arm, and leg without cortical symptoms [1]. CWS is usually associated with a high risk of capsular infarction with permanent deficits. A large population-based study of CWS has found that CWS is rare (1.5% of TIA presentations) but has a poor prognosis (7-day stroke risk of 60%) [6]. Our two cases both had subsequent capsular strokes after several episodes of TIAs, which are consistent with previous reports.

The exact pathogenic mechanism of CWS has not been fully understood. Various mechanisms have suggested, including small vessel disease, embolism from the heart, vasospasm, peri-infarct depolarization, and, in rare instances, atherosclerotic disease of the MCA [1,7-10]. Small perforator artery disease is proposed to be the most common cause of the CWS [1,10,11]. Recently, more studies suggested that intracranial atherosclerotic disease plays an important role in the development of small stratiocapsular infarct, especially in Asian [12]. Atherosclerotic plaque of the MCA may occlude the origin of the lenticulostriate arteries resulting in hemodynamic compromise and subsequent infarct. So we suppose that artherosclerotic disease of the MCA may be an important and common pathogenic mechanism of CWS. However, based on the current traditional imaging technique, little is known about the role of atherosclerotic disease in the development of the CWS. Recently, the technique of HR MRI has been developed to depict intracranial artery wall in vivo [4,5]. HR MR imaging is able to image a small plaque that did not yield stenosis on MRA [13,14]. In our cases, eccentric small plaques on the ventral wall of proximal MCA were identified on HR MRI [15]. Detection of these small lesions may carry clinical import because a non-stenostic plaque potentially causes ischemic symptoms via occlusion the origin of the lenticulostriate arteries. The fluctuating course of stereotyped symptoms was thought to be the result of hemodynamic compromise due to the origin occlusion. Relying on the HR MRI findings, we suggest that artherosclerotic disease of the MCA is an important pathophysiology of CWS.

Different treatment modalities, including hydration, antiplatelet agents, intravenous thrombolysis and intracranial artery angioplasty, have been proposed in patients with CWS [1,8,9,11,16]. It remains a challenge to develop therapies that may prevent irreversible damage to occur in patients with CWS. It seems aggressive medical treatments (including dual antithrombotic agents of aspirin and clopidogrel, and statin) might be effective on prevention the recurrent stroke in our cases.

## Conclusion

In summary, Our HR MRI data offer an insight into the pathophysiology of CWS which might be caused from atherosclerotic plaque in non-stenotic MCA wall. Early recognition of this clinical presentation and the accompanying stroke mechanism may guide the initial management and prognosis. Further studies using HR MRI in larger sample subjects are expected for highlighting this mechanism and guiding the acute treatment decision for CWS patients.

## Consent

“Written informed consent was obtained from the patient for publication of this case report and any accompanying images. A copy of the written consent is available for review by the Series Editor of this journal”.

## Competing interests

The authors declare that they have no competing interests.

## Authors’ contributions

LZ and JN were responsible for writing the article. MY was responsible for collecting case information. ML was responsible for collecting and analyzing imaging data. WX, BP and LC were responsible for revising the article. All authors read and approved the final manuscript.

## Pre-publication history

The pre-publication history for this paper can be accessed here:

http://www.biomedcentral.com/1471-2377/14/16/prepub
